# Supplementary material for: The cost of obtaining rewards enhances the reward prediction error signal of midbrain dopamine neurons
Source: Nat Commun. 2019 Aug 15;10:3674. doi: 10.1038/s41467-019-11334-2 (PMC6695452; doi:10.1038/s41467-019-11334-2)
Supplement: Supplementary file 1 — Supplementary Information [file 41467_2019_11334_MOESM1_ESM.docx]

# Supplementary Information

**The cost of obtaining rewards enhances the reward prediction error signal of midbrain dopamine neurons**

Tanaka et al.


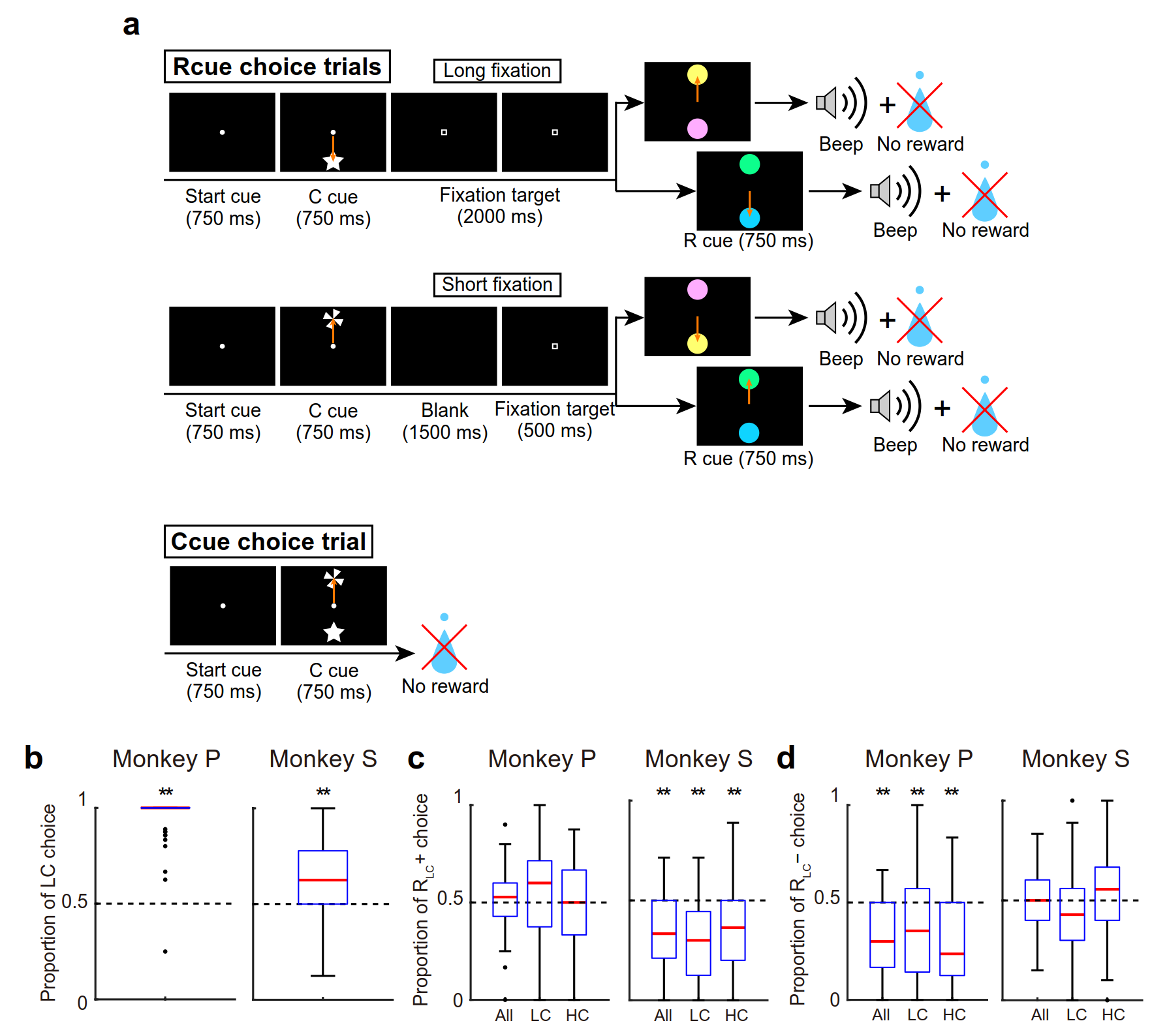


**Supplementary Fig. 1 Choice trials in the HLC saccade task**

(a) Choice trials in the HLC task. (b) The proportion of LC choice in the choice trials between the HC and LC cues (***P* < 0.01, two-tailed *t*-test; *t*_57_ = 28.3, *P* ≈ 0, n = 58 for monkey P; *t*_62_ = 4.9, *P* = 6.9 × 10^−6^, n = 63 for monkey S). In box-and-whisker plots, the central blue rectangle spans the first quartile to the third quartile and the red segment inside the rectangle denotes the median; the ends of the whiskers represent the minimum and maximum of the data, excluding outliers (denoted by black dots). (c) The proportion of R_LC_+ choice in the choice trials between R_HC_+ and R_LC_+ cues. LC and HC indicate choice after the short and long fixation, respectively. All indicates the mean of HC and LC. Monkey P showed no overt preference between R_HC_+ and R_LC_+ cues (two-tailed *t*-test; *t*_57_ = 1.1, *P* = 0.26, n = 58) but monkey S preferentially chose the R_HC_+ cue when he performed the choice task between R_HC_+ and R_LC_+ cue (two-tailed *t*-test; *t*_62_ = 8.6, *P* = 3.6 × 10^−12^, n = 63). (d) Proportion of the R_LC_− choice in the choice trials between R_HC_− and R_LC_− cues. Monkey P showed a preference to the R_HC_− cue when he performed the choice task between R_HC_− and R_LC_− cues (two-tailed *t*-test; *t*_57_ = 7.3, *P* = 8.5 × 10^−10^, n = 58) but monkey S showed no preference between R_HC_− and R_LC_− cues (two-tailed paired *t*-test; *t*_62_ = 0.54, *P* = 0.59, n = 63).


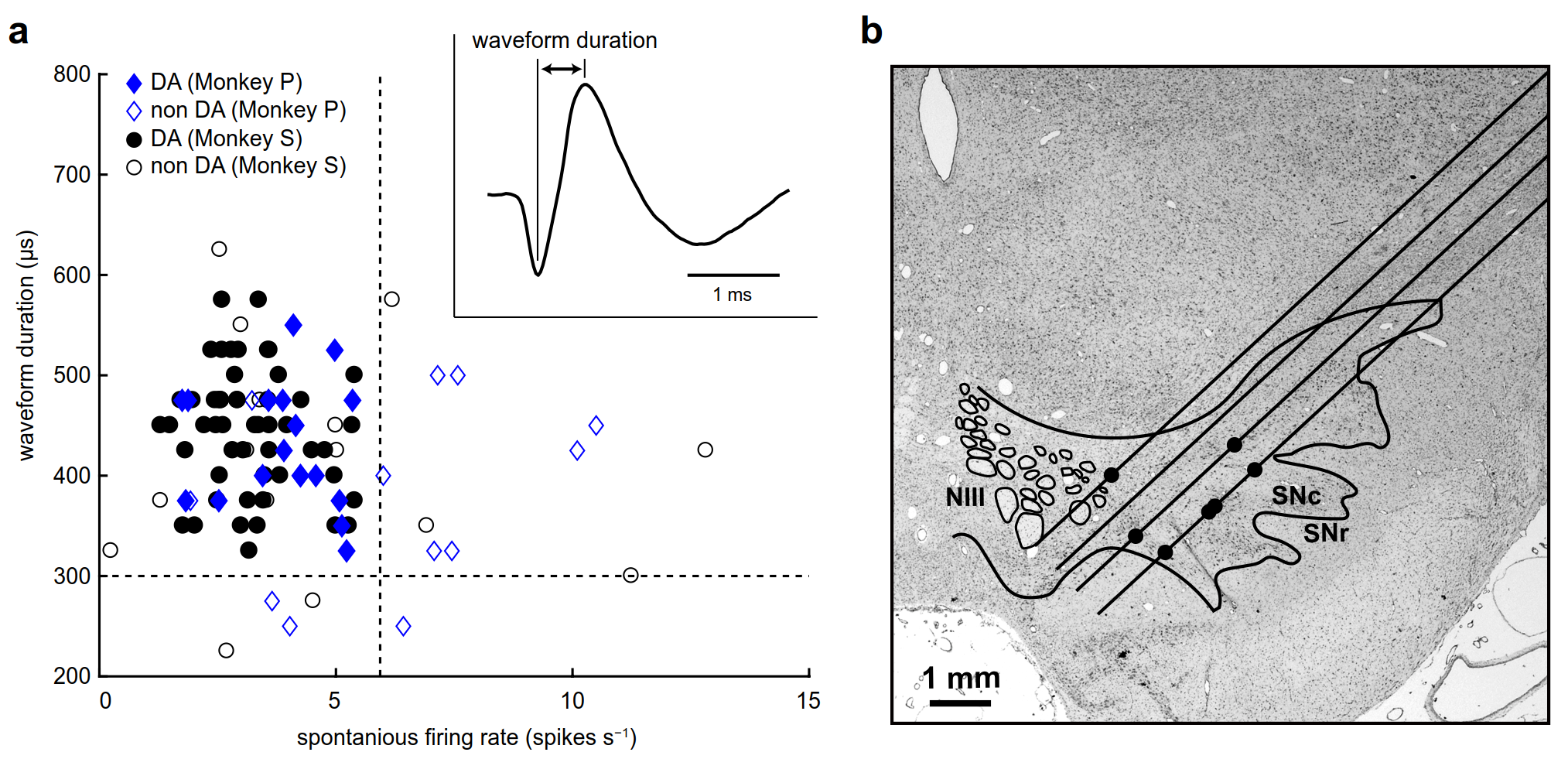


**Supplementary Fig. 2 Identification of the dopamine neurons and the recording sites**

(a) The distribution of the spontaneous firing rates and the waveform length of the recorded neurons. Inset represents an example waveform from a putative dopamine neuron. (b) A representative image of the recording sites with Nissl staining from the left hemisphere of the monkey P. Tilted lines indicate electrode tracks. Filled circles indicate the recording sites of putative dopamine neurons. SNc, Substantia nigra pars compacta; SNr, Substantia nigra pars reticulata; NIII, oculomotor nerve outlets.


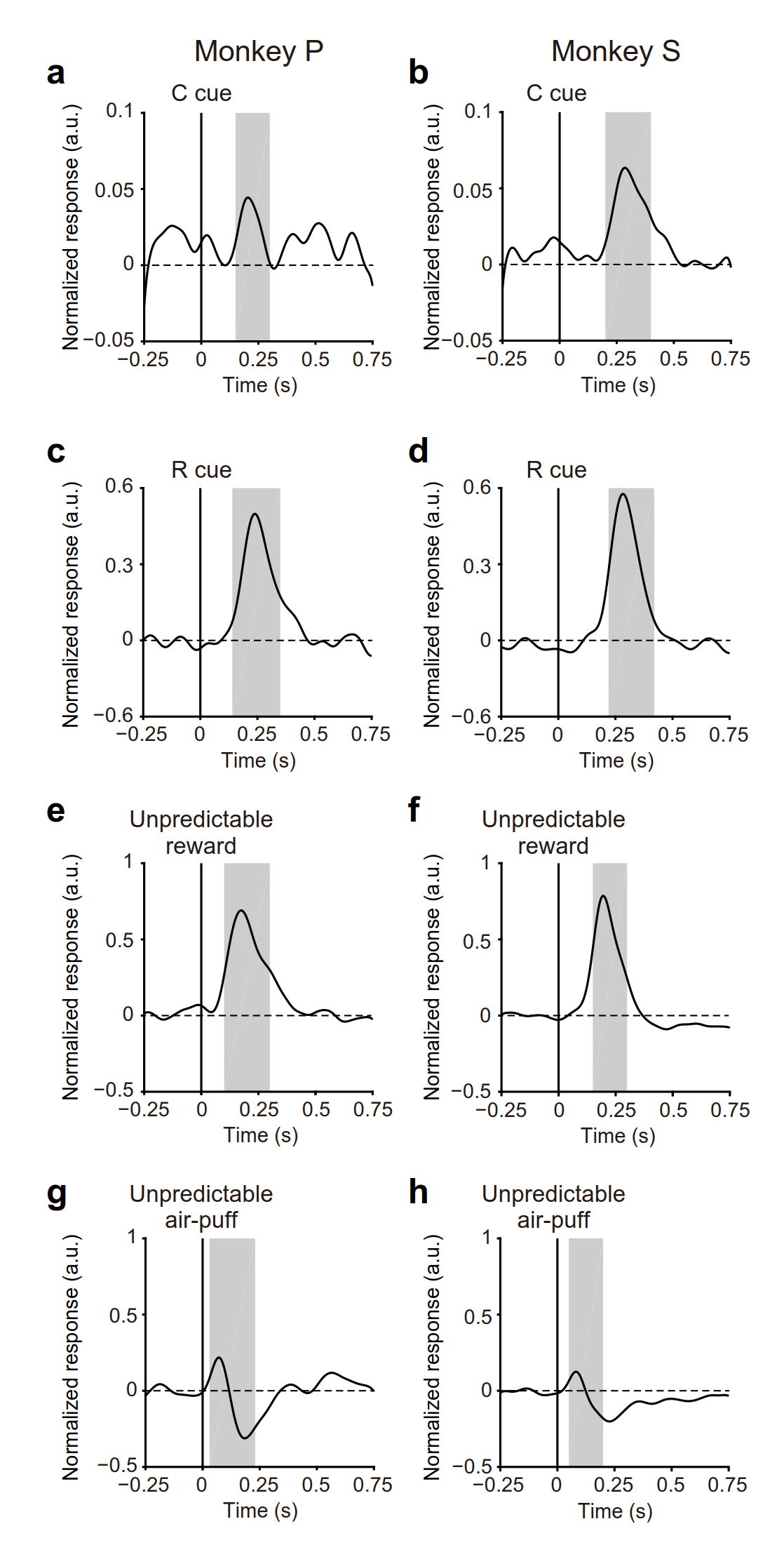


**Supplementary Fig. 3 Population averaged response of dopamine neurons and time windows**

(a, b) Population averaged response of the dopamine neurons to the cost cues recorded from monkey P (a) and S (b). The vertical line indicates the timing of the cost cue presentation. Gray-colored area indicates the time window to calculate the firing rate as the response to the condition cues. (c, d) Differences between the population averaged responses of the dopamine neurons to the reward (R_HC_+, R_LC_+) and no reward (R_HC_−, R_LC_−) cues, recorded from monkey P (c) and S (d). (e, f) Population averaged responses of the dopamine neurons to the unpredictable reward recorded from monkey P (e) and S (f). (g, h) Population averaged responses of the dopamine neurons to the unpredictable air-puff recorded from monkey P (g) and S (h).


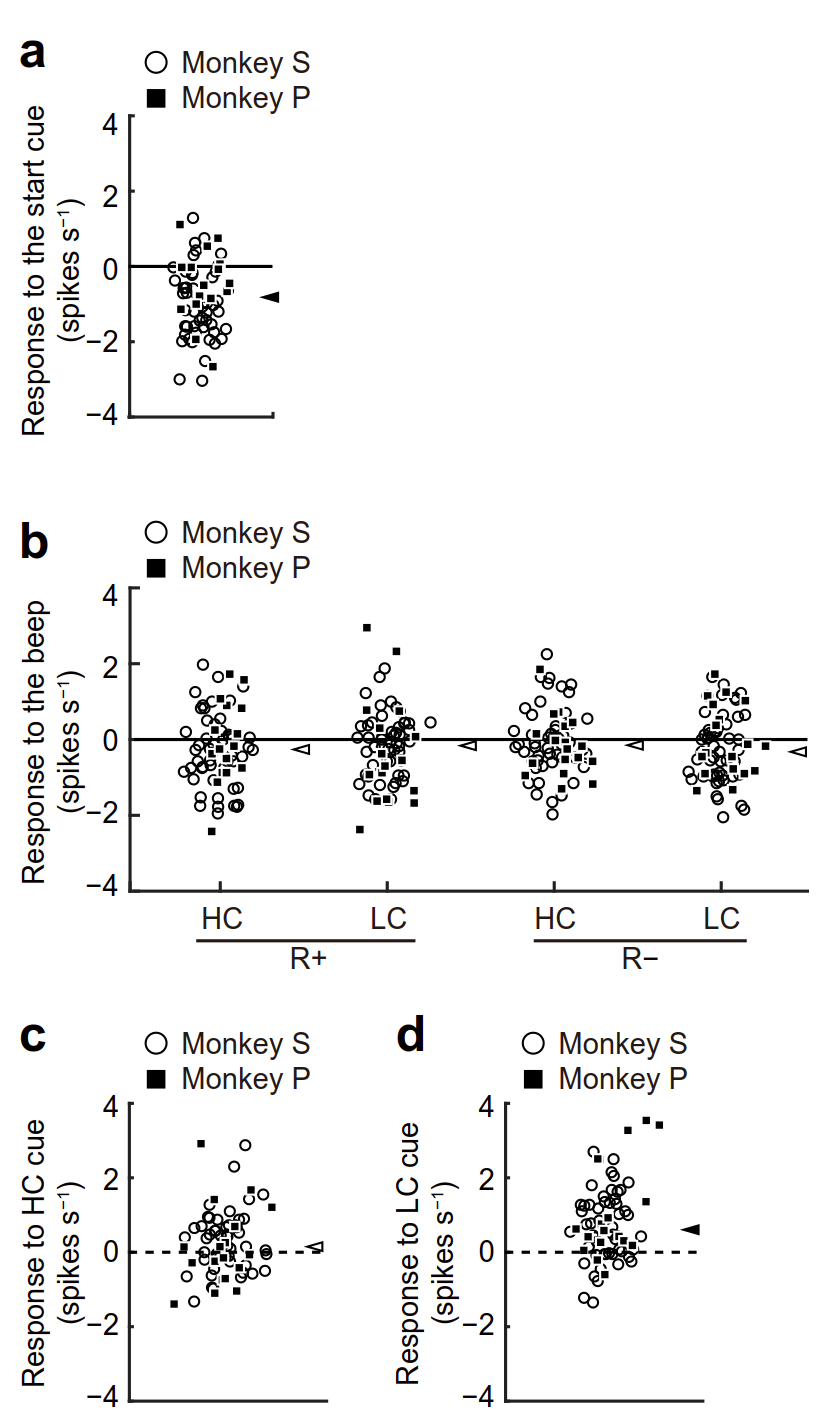


**Supplementary Fig. 4 Dopamine activity in the HLC saccade task**

(a) Distribution of the responses to the start cue relative to the spontaneous activity. Filled squares and open circles indicate data from monkey P and S, respectively. The arrowhead indicates the median of the response (−0.82). Across the population of dopamine neurons, the evoked response was smaller than 0 (two-tailed Wilcoxon’s signed-rank test, *P* = 3.0 × 10^−9^, *n* = 70). (b) Distribution of the responses to the beep sound (reward delivery) relative to the spontaneous activity. The arrowhead indicates the median of the response (HC R+: −0.26; LC R+: −0.63; HC R−: −0.15; LC R−: −0.35). The dopamine neurons did not show the significant response (two-tailed Wilcoxon’s signed-rank test, HC R+: *P* = 0.086; LC R+: *P* = 0.11; HC R−: *P* = 0.25; LC R−: *P* = 0.13; *n* = 70). (c) Distribution of the responses to the HC cue relative to the spontaneous activity. The arrowhead indicates the median of the response (0.15). The dopamine neurons did not show a significant response (two-tailed Wilcoxon’s signed-rank test, *P* = 0.56, *n* = 70). (d) Distribution of the responses to the LC cue relative to the spontaneous activity. The arrowhead indicates the median of the response (0.60). Across the population of dopamine neurons, the evoked response was larger than 0 (two-tailed Wilcoxon’s signed-rank test, *P* = 1.5 × 10^−7^, *n* = 70).


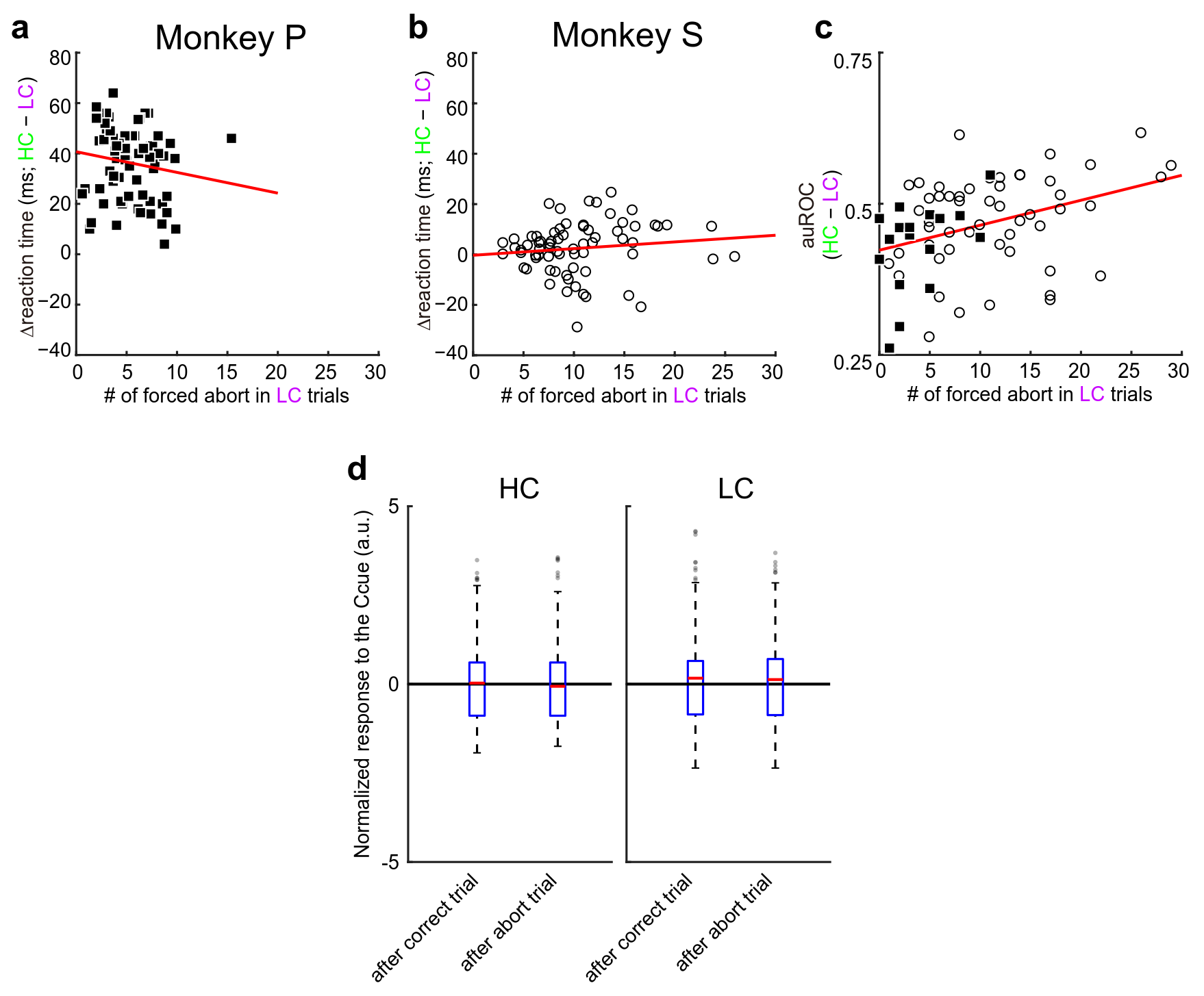


**Supplementary Fig. 5 Effect of the forced abort on the behavioral response and activity of the dopamine neurons to the C cues**

If the risk of the trial being aborted is increased by the forced aborts and if this caused the change in valuation and enhanced activation to the LC cue, as the number of forced aborts increase, the monkeys should value the LC cue more and the dopamine neurons should respond more to the LC cue.

(a) The relationship between the number of the forced abort in the low-cost trials and the difference between the reaction time to the high-cost cue and the low-cost cue of Monkey P calculated for each experimental day. The average number of the forced abort and the average reaction time in the experimental session of the HLC saccade task were calculated for each experimental day. There was no significant correlation (Pearson’s correlation coefficient, *r* = −0.15, *P* = 0.22, n = 68). The red line indicates the least squares regression line. (b) The relationship between the number of the forced abort in the low-cost trials and the difference between the reaction time to the high-cost cue and the low-cost cue of Monkey S calculated for each experimental day. There was no significant correlation (Pearson’s correlation coefficient, *r* = 0.12, *P* = 0.31, n = 70). (c) The relationship between the number of the forced aborts in the low-cost trials and the auROC between the dopamine responses to the high-cost cue and the low-cost cue. The dopamine responses were calculated for each dopamine neuron and the numbers of the forced aborts were calculated for the recording sessions of the HLC saccade task for each dopamine neuron. If the number of forced aborts increased activation to the LC cue, we would expect to find a negative correlation between the number of forced aborts and the auROC. On the contrary, there was a significant positive correlation (Pearson’s correlation coefficient, *r* = 0.37, *P* = 0.0018, n = 70). (d) The distribution of the normalized dopamine responses (z-scores) to the C cues after correct trials and those after abort trials. There was no significant difference between the response of the dopamine neuron to the cost cues after correct trials and after abort trials in high-cost condition (two-tailed two-sample *t*-test; *t*_3519_ = 0.99, *P* = 0.32; after correct trial: mean = −0.049, n = 2771; after abort trial: mean = −0.085, n = 1449) and low-cost condition (two-tailed two-sample *t*-test; *t*_3684_ = 1.32, *P* = 0.18; after correct trial: mean = 0.73, n = 2777; after abort trial: mean = 0.26, n = 1417).


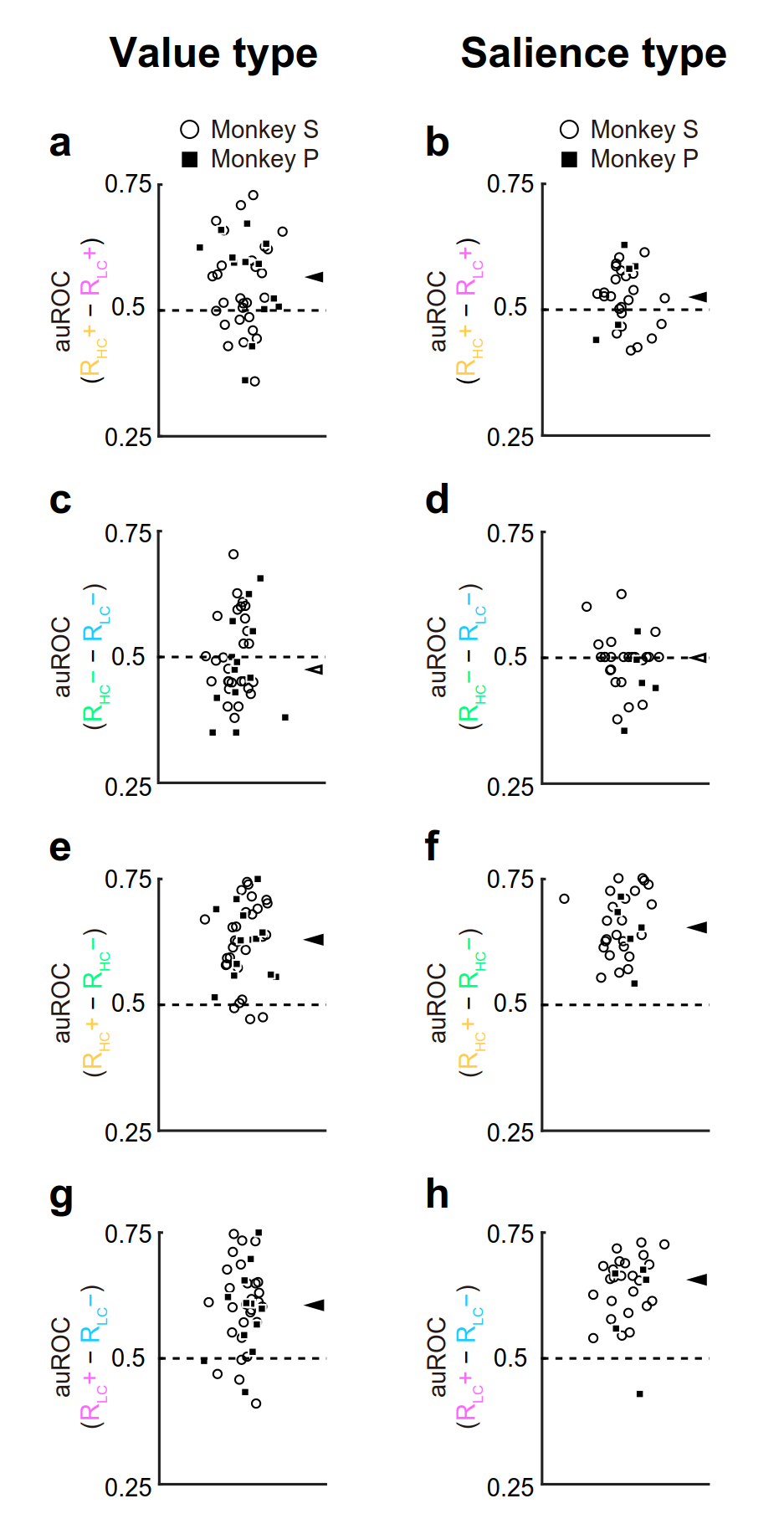


**Supplementary Fig. 6 Effect of the paid cost on the response to reward cues in value and salience type neurons**

Both types of dopamine neurons showed enhanced responses to the R+ cue in the high-cost condition than in the low-cost condition and the responses to the R− cues were not affected by the paid cost.

(a) The distribution of the auROCs calculated from the response of the value type neurons to the R+ cues in the high-cost and low-cost trials. The arrowhead indicates the median of the auROC (0.57). This was significantly larger than 0.5 (two-tailed Wilcoxon’s signed-rank test, *P* = 0.0013, *n* = 41). (b) The distribution of the auROCs calculated from the response of the salience type neurons to the R+ cues in the high-cost and low-cost trials. The arrowhead indicates the median of the auROC (0.53), which was significantly larger than 0.5 (two-tailed Wilcoxon’s signed-rank test, *P* = 0.043, *n* = 29). (c) The distribution of the auROCs calculated from the response of the value type neurons to the R− cues in the high-cost and low-cost trials. The arrowhead indicates the median of the auROC (0.48), which was not significantly different from 0.5 (two-tailed Wilcoxon’s signed-rank test, *P* = 0.72, *n* = 41). (d) The distribution of the auROCs calculated from the response of the salience type neurons to the R− cues in the high-cost and low-cost trials. The arrowhead indicates the median of the auROC (0.50) which was not significantly different from 0.5 (two-tailed Wilcoxon’s signed-rank test, *P* = 0.28, *n* = 29). (e) Distribution of the auROCs calculated from the response of the value type neurons to the R+ and R− cues in the high-cost trials. The arrowhead indicates the median of the auROC (0.76) which was significantly larger than 0.5 (two-tailed Wilcoxon’s signed-rank test, *P* = 6.3 × 10^−8^, *n* = 41). (f) The distribution of the auROCs calculated from the response of the salience type neurons to the R+ and R− cues in the high-cost trials. The arrowhead indicates the median of the auROC (0.81) which was significantly larger than 0.5 (two-tailed Wilcoxon’s signed-rank test, *P* = 2.6 × 10^−6^, *n* = 29). (g) The distribution of the auROCs calculated from the response of the value type neurons to the R+ and R− cues in the low-cost trials. The arrowhead indicates the median of the auROC (0.71) and significantly biased to be larger than 0.5 (two-tailed Wilcoxon’s signed-rank test, *P* = 4.8 × 10^−7^, *n* = 41). (h) The distribution of the auROCs calculated from the response of the salience type neurons to the R+ and R− cues in the low-cost trials. The arrowhead indicates the median of the auROC (0.81) and significantly biased to be larger than 0.5 (two-tailed Wilcoxon’s signed-rank test, *P* = 4.3 × 10^−6^, *n* = 29).


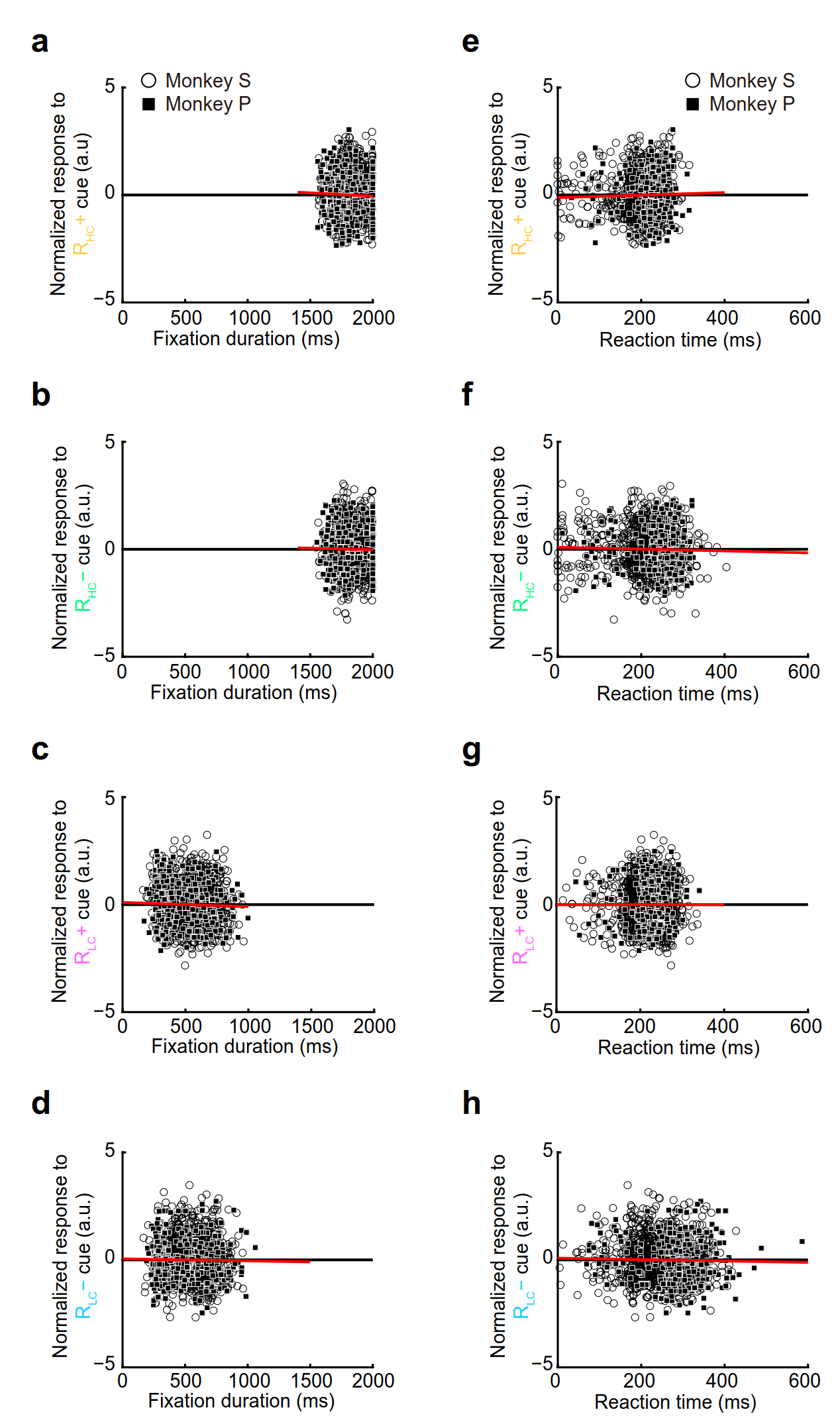


**Supplementary Fig. 7 Relationship between the actual fixation duration or the reaction time and the dopamine response**

(a) The relationship between the fixation duration and the normalized dopamine responses (z-scores) to the R_HC_+ cue. Filled squares and open circles indicate data from monkey P and S, respectively. Red line represents a least squares regression line. There was no significant correlation (Pearson’s correlation coefficient, *r* = −0.031, *P* = 0.24, n = 1400). (b) The relationship between the fixation duration and the normalized dopamine responses (z-scores) to the R_HC_− cue. There was no significant correlation (Pearson’s correlation coefficient, *r* = −0.016, *P* = 0.54, n = 1400). (c) The relationship between the fixation duration and the normalized dopamine responses (z-scores) to the R_LC_+ cue. There was no significant correlation (Pearson’s correlation coefficient, *r* = −0.032, *P* = 0.23, n = 1400). (d) The relationship between the fixation duration and the normalized dopamine responses (z-scores) to the R_LC_− cue. There was no significant correlation (Pearson’s correlation coefficient, *r* = −0.016, *P* = 0.55, n = 1400). (e) The relationship between the reaction times and the normalized dopamine responses (z-scores) to the R_HC_+ cue. There was no significant correlation (Pearson’s correlation coefficient, *r* = 0.028, *P* = 0.29, n = 1400). (f) The relationship between the reaction times and the normalized dopamine responses (z-scores) to the R_HC_− cue. There was no significant correlation (Pearson’s correlation coefficient, *r* = −0.027, *P* = 0.31, n = 1400). (g) The relationship between the reaction times and the normalized dopamine responses (z-scores) to the R_LC_+ cue. There was no significant correlation (Pearson’s correlation coefficient, *r* = −0.00014, *P* = 0.99, n = 1400). (h) The relationship between the reaction times and the normalized dopamine responses (z-scores) to the R_HC_− cue. There was no significant correlation (Pearson’s correlation coefficient, *r* = −0.024, *P* = 0.38, n = 1400).


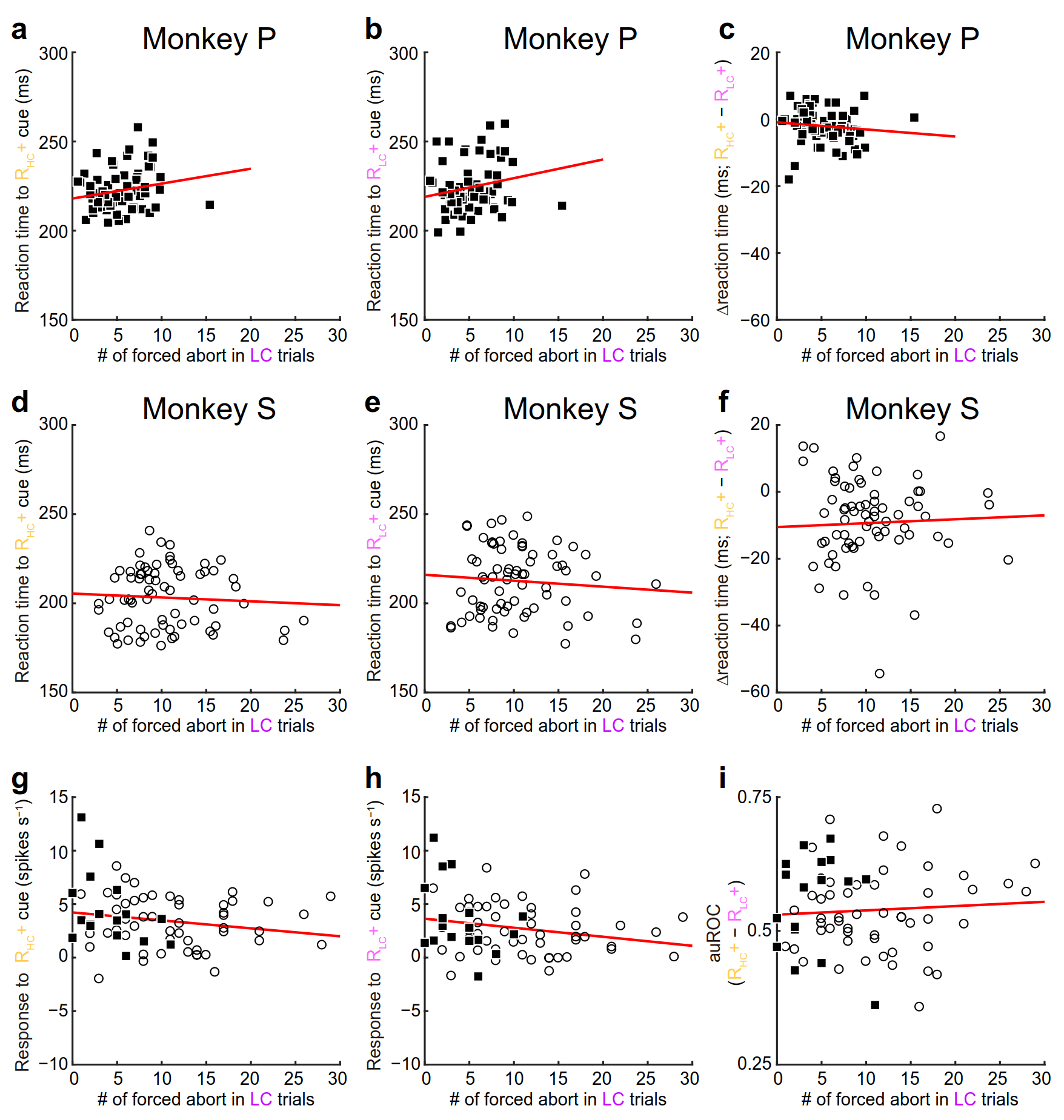


**Supplementary Fig. 8 Effect of the forced abort on the behavioral response and activity of the dopamine neurons to the R cues**

(a) The relationship between the number of the forced abort in the low-cost condition and the reaction time to the R_HC_+ cue of Monkey P. The average number of the forced abort and the average reaction time in the experimental session of the HLC saccade task were calculated for each experimental day. There was no significant correlation (Pearson’s correlation coefficient, *r* = 0.20, *P* = 0.10, n = 68). The red line indicates the least squares regression line. (b) The relationship between the number of the forced abort in the low-cost condition and the reaction time to the R_LC_+ cue of Monkey P calculated for each experimental day. There was no significant correlation (Pearson’s correlation coefficient, *r* = 0.20, *P* = 0.095, n = 68). (c) The relationship between the number of the forced abort in the low-cost condition and the difference between the reaction time to the R_HC_+ cue and the R_LC_+ cue of Monkey P calculated for each experimental day. There was no significant correlation (Pearson’s correlation coefficient, *r* = −0.12, *P* = 0.34, n = 68). (d) The relationship between the number of the forced abort in the low-cost condition and the reaction time to the R_HC_+ cue of Monkey S calculated for each experimental day. There was no significant correlation (Pearson’s correlation coefficient, *r* = −0.062, *P* = 0.61, n = 70). (e) The relationship between the number of the forced abort in the low-cost condition and the the reaction time to the R_LC_+ cue of Monkey S calculated for each experimental day. There was no significant correlation (Pearson’s correlation coefficient, *r* = −0.087, *P* = 0.47, n = 70). (f) The relationship between the number of the forced abort in the low-cost condition and the difference between the reaction time to the R_HC_+ cue and the R_LC_+ cue of Monkey S calculated for each experimental day. There was no significant correlation (Pearson’s correlation coefficient, *r* = −0.041, *P* = 0.74, n = 70). (g) The relationship between the number of the forced abort in the low-cost condition and the dopamine responses to the R_HC_+ cue calculated for each experimental day. The dopamine responses were calculated for each dopamine neurons and the numbers of the forced abort were calculated for each recording session of the HLC saccade task. There was no significant correlation (Pearson’s correlation coefficient, *r* = −0.20, *P* = 0.097, n = 70). (h) The relationship between the number of the forced abort in the low-cost condition and the dopamine responses to the R_LC_+ cue calculated for each dopamine neuron recording. There was no significant correlation (Pearson’s correlation coefficient, *r* = −0.23, *P* = 0.055, n = 70). (i) The relationship between the number of the forced abort in the low-cost condition and auROC between the dopamine responses to the R_HC_+ cue and the R_LC_+ cue calculated for each dopamine neuron recording. There was no significant correlation (Pearson’s correlation coefficient, *r* = 0.045, *P* = 0.71, n = 70).


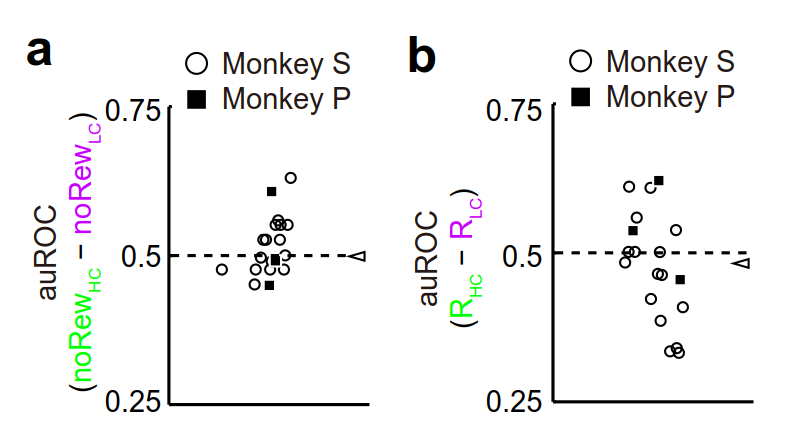


**Supplementary Fig. 9 Dopamine activity in the HLC uncertain task**

(a) The distribution of the auROCs calculated from the responses to the no reward delivery in the high-cost and low-cost trials. The arrowhead indicates the median of the auROC (0.50) which showed no significant deviatin from 0.5 (two-tailed Wilcoxon’s signed-rank test, *P* = 0.31, *n* = 19). (b) The distribution of the auROCs calculated from the responses to the R cues in the high-cost and low-cost trials. The arrowhead indicates the median of the auROC (0.48) which showed no significant deviation from 0.5 (two-tailed Wilcoxon’s signed-rank test, *P* = 0.33, *n* = 19).


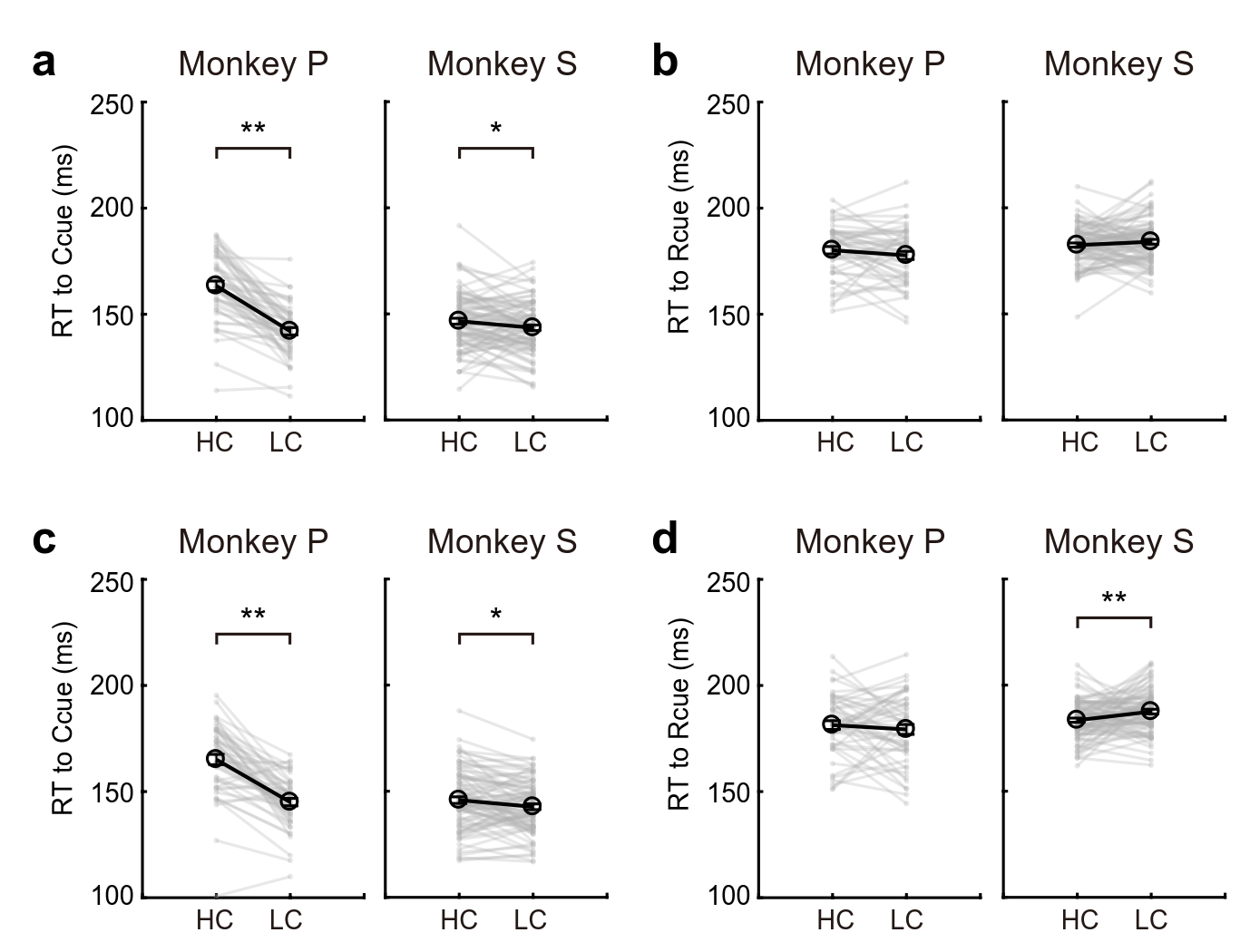


**Supplementary Fig. 10 Behavioral data in the HLC exploration task**

(a) Reaction times to cost cues in the first half of the HLC exploration task. The monkeys showed faster reaction times to the low-cost cue (**P* < 0.05; ***P* < 0.01, two-tailed paired *t*-test). Black circles and error bars indicate mean and SEM. (b) Reaction times to the reward cues in the first half of the HLC exploration task. (c) The reaction time to the cost cues in the latter half of the HLC exploration task. The monkeys showed faster reaction times to the low-cost cue (**P* < 0.05; ***P* < 0.01, two-tailed paired *t*-test). (d) Reaction times to the reward cues in the latter half of the HLC exploration task. Monkey S showed faster reaction times to the reward cues in the high-cost condition (***P* < 0.01, two-tailed paired *t*-test).


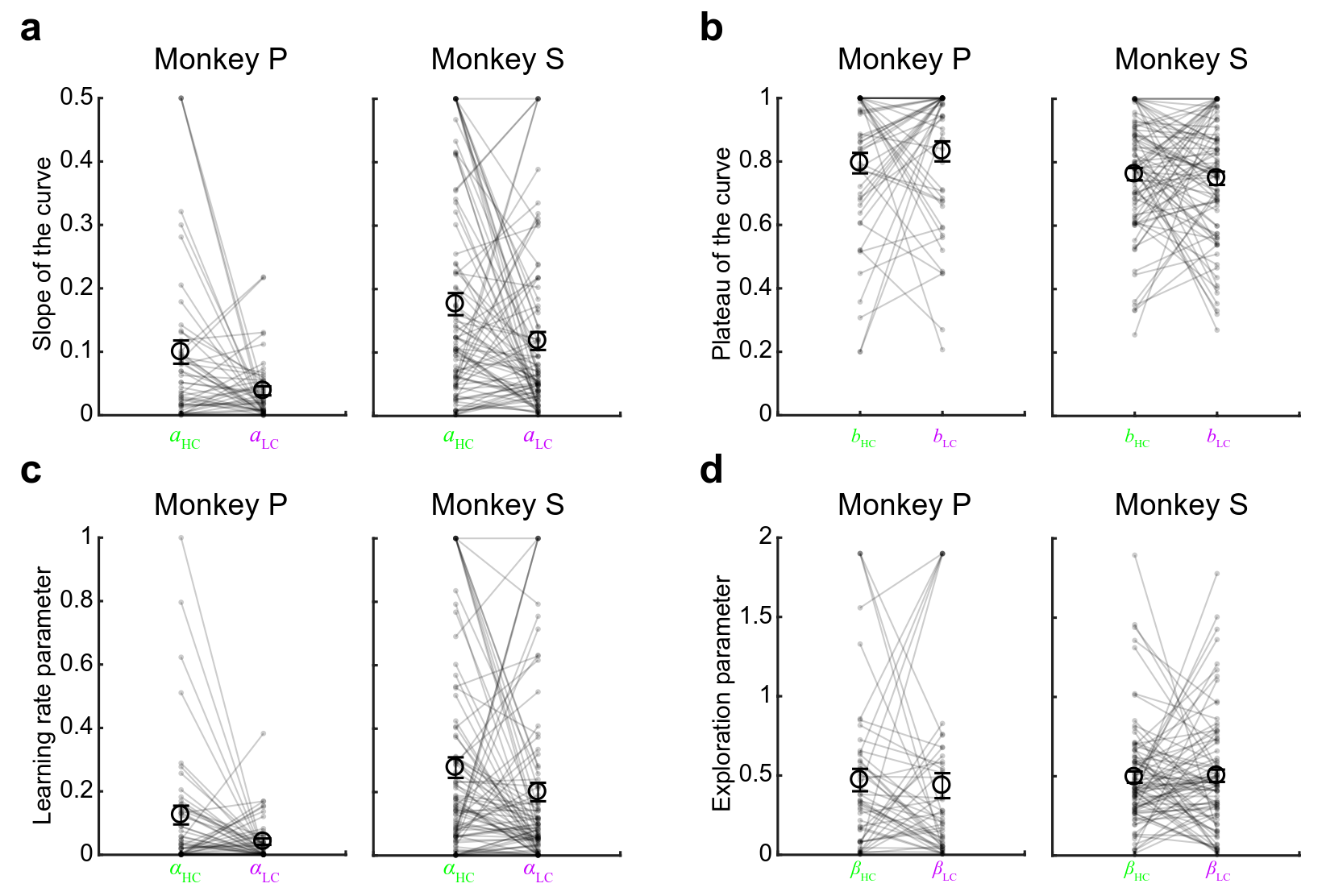


**Supplementary Fig. 11 Fitting parameters in the learning speed estimation**

(a) The fitting parameters *a* in the high- and low-cost conditions when the data were fit with a cumulative exponential function. Black circles and error bars indicate mean and SEM. (b) The fitting parameters *b* in the high- and low-cost conditions when the data were fit with a cumulative exponential function. (c) The learning rate parameter *α* in the high- and low-cost conditions when the data were fit with a reinforcement learning model. (d) The fitting parameter *β* in the high- and low-cost conditions when the data were fit with a reinforcement learning model.


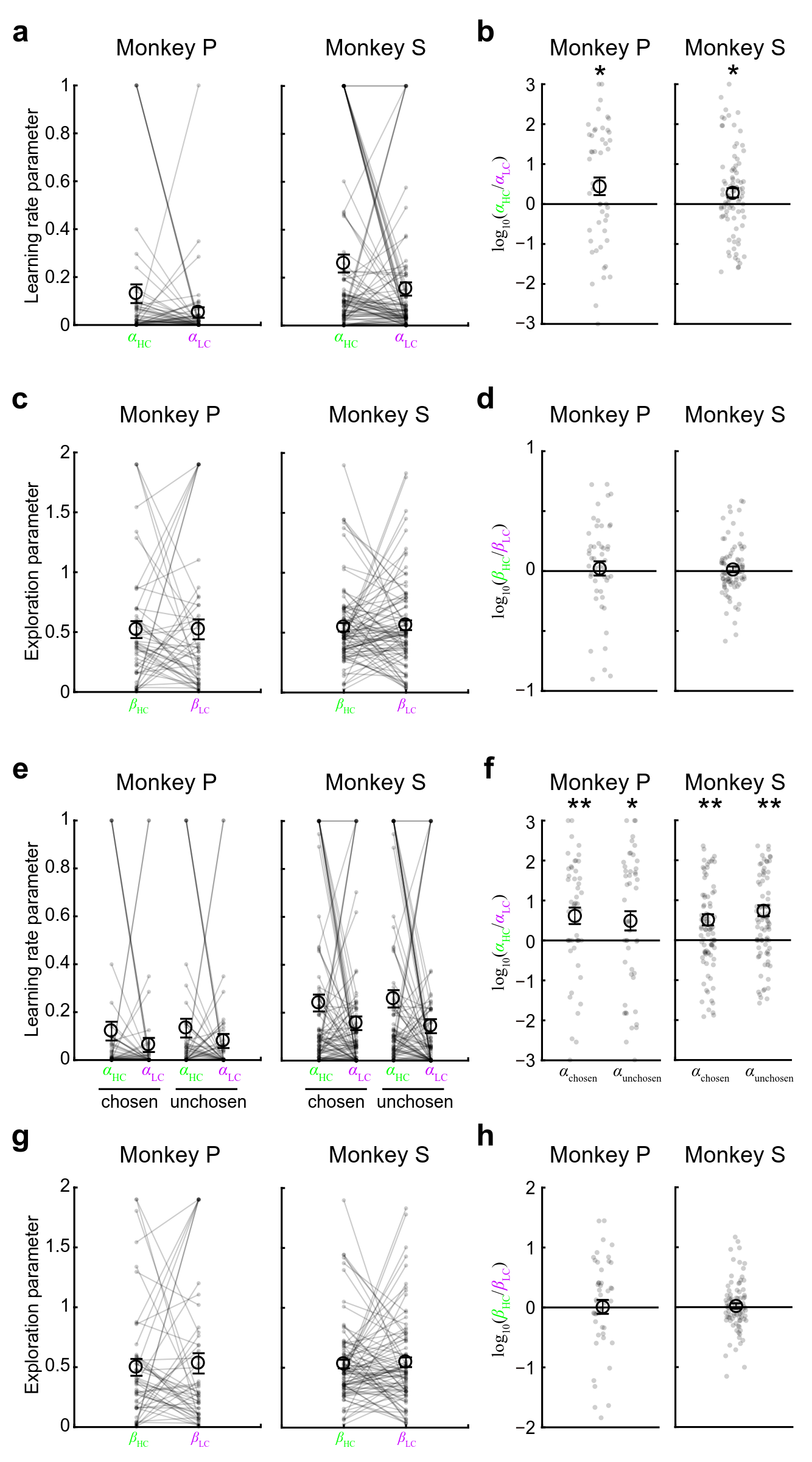


**Supplementary Fig. 12 Learning speed estimation with alternative RL models that update unchosen and chosen action values**

Here we describe the results from fitting two additional RL models to the data. Unlike the model used for the analysis reported in Fig. 8 in the main manuscript, in these models both the chosen option and unchosen option were updated based on an anticorrelation in the reward structure. One of the models (panels a - d) utilized the same learning rate parameter for updating the chosen and unchosen option. The other model (panels e - f) utilized independent learning rate parameter for the chosen and unchosen options.

(a) The learning rate parameters *α* in the high- and low-cost conditions when the data were fit with a shared learning rate for chosen and unchosen actions. Black circles and error bars indicate mean and SEM. (b) The log ratio between the learning rate parameters *α* in the high- and low-cost conditions when the data were fit with a shared learning rate for chosen and unchosen actions (**P* < 0.05; two-tailed *t*-test). The log ratio between the learning rate parameters was significantly larger than zero (two-tailed *t*-test; *t*_48_ = 2.0 *P* = 0.049, mean = 0.44, *n* = 49 for monkey P; *t*_85_ = 2.4, *P* = 0.017, mean = 0.28, *n* = 86 for monkey S). (c) The exploration parameter *β* in the high- and low-cost conditions. (d) The log ratio between the exploration parameter *β* in the high- and low-cost conditions. The log ratio between the exploration parameters did not significantly biased from zero (two-tailed *t*-test; *t*_48_ = 0.37, *P* = 0.72, mean = 0.043, *n* = 49 for monkey P; *t*_85_ = 0.56, *P* = 0.58, mean = 0.030, *n* = 86 for monkey S). (e) The learning rate parameters *α* in the high- and low-cost conditions when the data were fit with a model in which the chosen and unchosen actions had separate learning rates (f) The log ratio between the learning rate parameters *α* in the high- and low-cost conditions when the data were fit with a model in which the chosen and unchosen actions had separate learning rates (**P* < 0.05, ***P* < 0.01; two-tailed Wilcoxon’s signed-rank test). The log ratio between the learning rate parameters for chosen option were significantly larger than zero (two-tailed *t*-test; *t*_48_ = 3.0, *P* = 4.4 × 10^−3^, mean = 0.62, *n* = 49 for monkey P; *t*_85_ = 3.6, *P* = 4.4 × 10^−4^, mean = 0.51, *n* = 86 for monkey S). The log ratio between the learning rate parameters for unchosen option were also significantly larger than zero (two-tailed *t*-test; *t*_48_ = 2.0, *P* = 0.048, mean = 0.49, *n* = 49 for monkey P; *t*_85_ = 5.3, *P* = 7.2 × 10^−7^, mean = 0.74, *n* = 86 for monkey S). (g) The exploration parameter *β* in the high- and low-cost conditions. (h) The log ratio between the exploration parameter *β* in the high- and low-cost conditions. The log ratio between the exploration parameters did not significantly biased from zero (two-tailed *t*-test; *t*_48_ = 0.075, *P* = 0.94, mean = 0.0087, *n* = 49 for monkey P; *t*_85_ = 0.44, *P* = 0.66, mean = 0.020, *n* = 86 for monkey S). These results show that our conclusions about increased learning rates in the high cost condition still hold even with these alternative model formulations.
